# Response to formal comment on Myhrvold (2016) submitted by Griebeler and Werner (2017)

**DOI:** 10.1371/journal.pone.0192912

**Published:** 2018-02-28

**Authors:** Nathan P. Myhrvold

**Affiliations:** Intellectual Ventures, Bellevue, Washington, United States of America; Perot Museum of Nature and Science, UNITED STATES

## Abstract

Griebeler and Werner offer a formal comment on Myhrvold, 2016 defending the conclusions of Werner and Griebeler, 2014. Although the comment criticizes several aspects of methodology in Myhrvold, 2016, all three papers concur on a key conclusion: the metabolism of extant endotherms and ectotherms cannot be reliably classified using growth-rate allometry, because the growth rates of extant endotherms and ectotherms overlap. A key point of disagreement is that the 2014 paper concluded that despite this general case, one can nevertheless classify dinosaurs as ectotherms from their growth rate allometry. The 2014 conclusion is based on two factors: the assertion (made without any supporting arguments) that the comparison with dinosaurs must be restricted only to extant sauropsids, ignoring other vertebrate groups, and that extant sauropsid endotherm and ectotherm growth rates in a data set studied in the 2014 work do not overlap. The Griebeler and Werner formal comment presents their first arguments in support of the restriction proposition. In this response I show that this restriction is unsupported by established principles of phylogenetic comparison. In addition, I show that the data set studied in their 2014 work does show overlap, and that this is visible in one of its figures. I explain how either point effectively invalidates the conclusion of their 2014 paper. I also address the other methodological criticisms of Myhrvold 2016, and find them unsupported.

## 1. Introduction

The Griebeler and Werner formal comment [1] on Myhrvold, 2016 [2], as well as the earlier 2014 work by Werner and Griebeler [3], are part of a debate that has been going on for at least 40 years about the metabolic status of dinosaurs and a possible relation of the maximum rate of growth in body mass to the metabolic class (endothermic or ectothermic) of extant animals. In the late 1970s, Case [4] did at comparative study of vertebrate growth rates and metabolism. In a second paper [5] he used these results to speculate about dinosaur metabolism (he had no actual dinosaur growth rate data). Case did not find a direct connection between growth rate and metabolism, but subsequent studies of dinosaur growth rates [6–13] referenced Case’s papers and erroneously implied that they offer a firm basis for determining dinosaur metabolism by comparing the growth-rate allometry of dinosaurs to that of extant endotherms and ectotherms.

Werner and Griebeler [3] advanced this debate by publishing the first detailed analysis of the possible link between growth rate and metabolism and its application to determining dinosaur metabolism. Shortly thereafter, an independent study with similar aims by Grady et al. [14] took a related but distinct approach. Despite both being inspired by Case, the two papers reached strikingly different conclusions. While Werner and Griebeler [3] concluded that dinosaurs are ectotherms, Grady et al. [14] proposed that dinosaur metabolism was sufficiently different from either ectothermy or endothermy to warrant coining the new term “mesothermy”, to describe a metabolic state intermediate between ectothermy and endothermy.

In a short comment [15] on Grady et al. and subsequently a larger paper [2] that also examined arguments advanced by Werner and Griebeler [3], I attempted to resolve these contradictory views. My analyses confirmed via different techniques the earlier conclusion of Werner and Griebeler [3] that the overlap in growth rates of extant vertebrates makes it infeasible to determine their metabolic status in this way. In addition to this important point of agreement, Greibeler and Werner [1] concur with Myhrvold [2] that Case [4,5] did not suggest that metabolism was “solely determined” by growth rate, and that he did not propose that metabolism could be determined by comparisons of growth-rate allometry (i.e., regressions of growth rate versus body mass). We further agree that the dinosaur growth-rate studies [6–13] that referenced Case failed to link maximum growth-rate allometry to metabolic status but rather implicitly assumed, incorrectly, that Case had established that link.

This response addresses the most important unresolved difference in our views as well as specific criticisms raised by Greibeler and Werner [1] regarding Myhrvold [2] and its methodology. Sections 2 and 3 discuss the important empirical question of whether the metabolic status of dinosaurs can be inferred by comparing their growth-rate allometry to that of extant animals. Werner and Griebeler [3] used such a comparison, restricted to sauropsids, to conclude that dinosaurs were ectotherms but offered no explanation for the arbitrary restriction. Their formal comment presents for the first time an argument for phylogenetic comparison to support this method. I show below that this argument is flawed; there is no valid reason to restrict the comparison to sauropsids; indeed there are powerful reasons not to do this.

Section 4 concerns the use of phylogenetic generalized least-squares regression (PGLS) in [3], which the formal comment [1] argues is superior to the ordinary least-squares regression (OLS), used in Myhrvold [2]. I show that the analysis method used in [3] causes the regression determined intercept to cancel out; the answer is the same regardless of whether PGLS or OLS was used to find the intercept.

Section 5 examines the empirical evidence claimed in [3] that the growth-rate allometry of extant endothermic and ectothermic sauropsids do not overlap. This is shown to be false–it is implicit in the data set and analysis of [3] and is even shown in one of its figures. Data from Grady et al. [14] shows additional overlap.

Sections 6, 7 cover criticisms in [1] regarding claims made in Myhrvold [2] about the use of the metabolic theory of ecology (MTE) in [3], and the relationship to two hypotheses framed in [2] about classification of metabolism by growth rate. Section 8 and 9 cover different choices for the dependent and independent variable in regression in [2] and [3], which were criticized in [1]. Section 10 deals with a criticism of [2] based on the method of adjusting metabolic rate to ambient temperature by Grady et al. [14], while section 11 covers the choice of vertebrate groups for comparison in [3]. Finally, section 12 is the conclusion.

## 2. Growth-rate allometry does not imply metabolism

Can we infer the metabolic status of an extant vertebrate species by comparing its growth rate and body size to growth rate and body size allometry of extant animals? Werner and Griebeler [3] concluded that the answer no: growth rates of extant endotherms and ectotherms overlap so much that they cannot be used to determine metabolism. On this we agree, as noted in Myhrvold [2] and the clarification in the comment [1].

As Werner and Griebeler have observed in the last paragraph of their paper [3]:

Our regression models revealed that dinosaurs had growth rates intermediate to similar sized/scaled-up reptiles and mammals, but had much lower rates than those observed in scaled-up birds. Furthermore, our results suggest that–irrespective of whether all dinosaurian growth rates were higher than that of an average reptile—they were still in the range of rates seen in fast growing reptiles. Dinosaurian rates are also consistent with those of slow growing mammals, but no dinosaur had a growth rate consistent with any precocial or altricial bird. Thus, under the assumption that growth rate, metabolic rate and thermoregulation are directly linked, it is not possible to infer whether the studied dinosaurs had an ectothermic or endothermic metabolic rate because of the large variability seen in ectothermic and endothermic growth rates.

The logic in the last sentence is convoluted. They state that if the assumption is *true* (i.e. “under the assumption”) that maximum growth rate is linked to metabolism, they conclude that we cannot infer anything about dinosaur metabolism. Yet the assumption being true would imply that one can infer metabolism, whereas the next sentence says that one cannot. That sentence is reaffirmed in the formal comment [1]. On the other hand, if the assumption is *false* we *also* cannot infer anything; if there is no link between maximum growth rate and metabolism, then we can’t use one to infer the other. So it is unclear what the phrase “under the assumption…” is contributing to the sentence.

Note that they refer to “the assumption” that growth rate and metabolic rate are linked is a proposition that their own results powerfully refute–i.e. because the growth rates of extant endotherms and ectotherms overlap one can *rule out a direct link*. The quote from the formal comment [1] above goes much further than listing it as an assumption–the authors confirm there is *no convincing evidence* for such a link.

One can classify a species as endothermic or exothermic by comparing maximum growth-rate allometry if and only if the maximum growth-rate allometry of extant endotherms and ectotherms are distinct; that is the operative definition of the “direct link” discussed above, at least with respect to the metabolic classification issue (see also below under H1 and H2).

Setting aside the strangely worded predicate, the quote above would appear to definitively answer the question. Had Werner and Griebeler ended their paper at the quote above, we would be in very substantial agreement. Unfortunately the very next sentence, which ends the paragraph above is this [3]

However, compared to growth rates seen in other sauropsids, all studied dinosaurs had rather an ectothermic metabolic rate than an endothermic rate.

This is the ultimate conclusion of the paper–the message that dinosaurs are ectotherms appears in both the title of the paper and its abstract. In that concluding sentence, and others preceding it in the paper which also reach that conclusion, they appear, in my view at least, to reverse themselves. How can it be that we cannot determine metabolism for extant groups, but could do it for dinosaurs? Why should the *only* point of comparison be sauropsids?

## 3. An unjustified restriction to sauropsids

**Fig 1:**
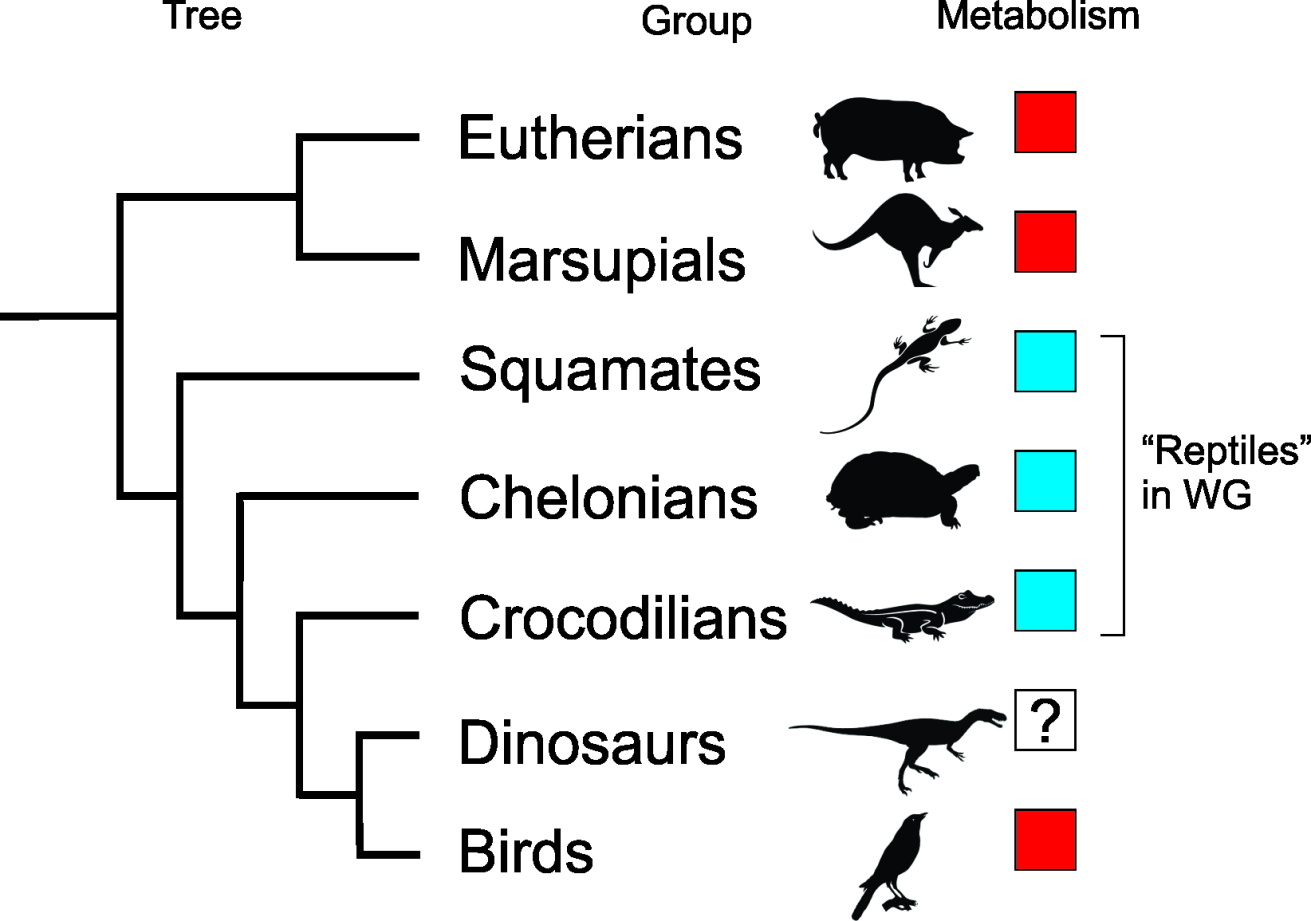
Simplified phylogenic tree. The phylogenetic relationship of extant groups [48] and dinosaurs relevant to Werner and Griebeler [3] highlights the endotherms (*red*) and the ectotherms (*blue*).

**Fig 2:**
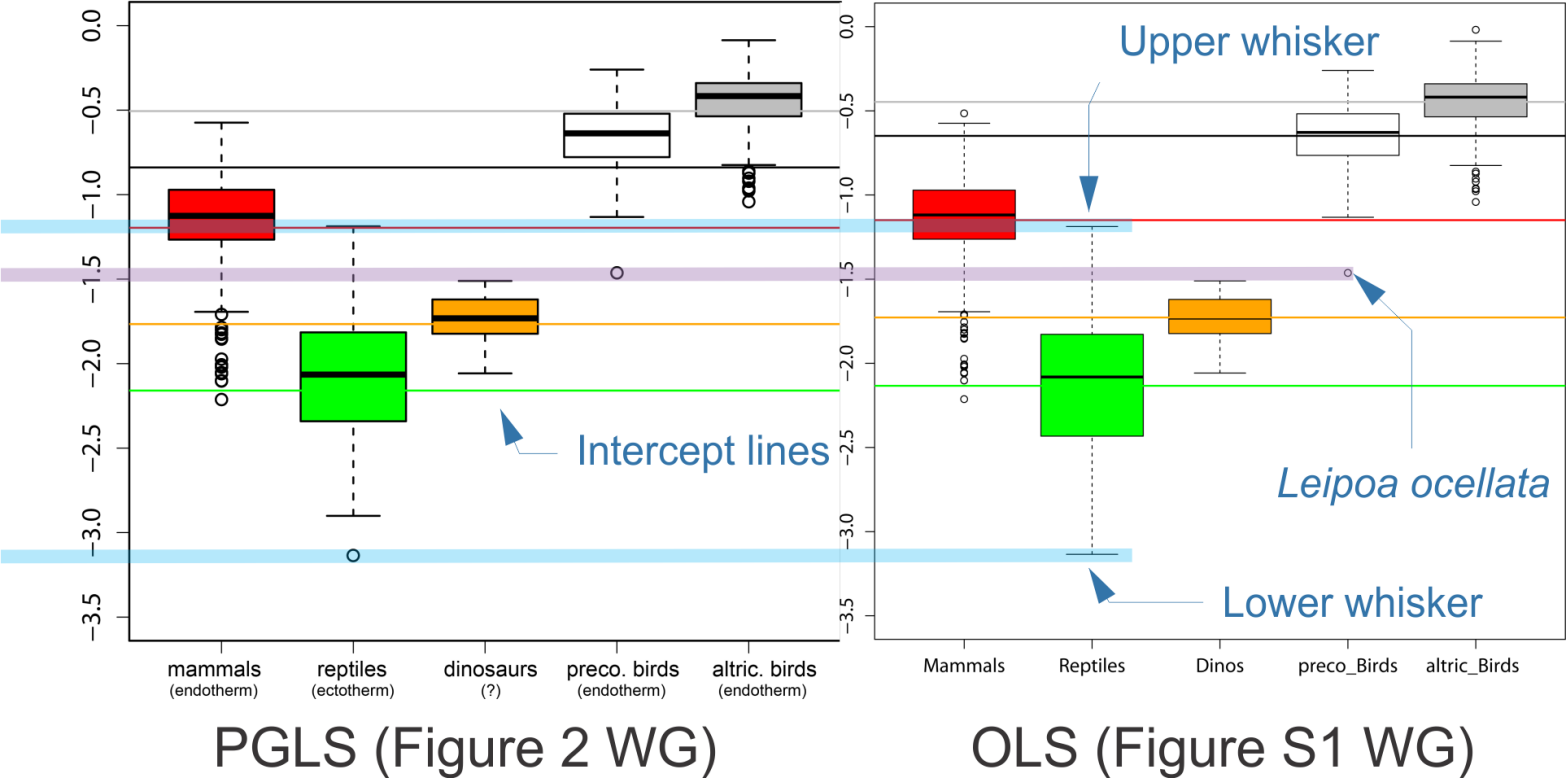
Demonstration that PGLS and OLS residual variation plots are nearly identical in the Werner and Griebeler study. As shown by Eq (3), the “residual variation” data plotted in Werner and Griebeler Fig 2 and S1 [3] is independent of whether it is based on PGLS or OLS regression. This is clearly seen here by reproducing Fig 2 and S1 of Werner and Griebeler [3] placed side by side, with added annotations (*blue* text and arrows, *blue* and *purple thick transparent lines*). The thin colored horizontal lines are plots of the OLS and PGLS intercepts; these differ between PGLS and OLS (as given by Tables 2, S3 of [3]–see also Fig 4 here) so these lines *are not the same in the two plots*. A thick blue transparent horizontal line has been overlaid on the plots to show that the upper whisker of the reptiles has identical position in the two plots. A purple transparent horizontal line shows that the position of the lowest precocial bird data point, *Leipoa ocellata* is also identical. As explained in [3], the OLS regressions include some taxa that are omitted from PGLS regressions because they lack necessary phylogenetic information. Despite this, the maximum range of each group, which determines the overlap that is crucial to the Werner and Griebeler analysis is identical.

The Werner and Griebeler study [3] does not justify the seeming contradiction between the sentences quoted above. The closest they come is this passage [3]:

Growth rate to body mass relations of dinosaurs are located within the intersection of the ectothermic and endothermic growth rates (Figs 1 and 2). Given this, the interpretation of endothermy or ectothermy in dinosaurs would be ambiguous. However, restricted to sauropsids, all studied non-avian dinosaurian growth rate to body mass relations were in the range of reptile growth rates and did not fit within those of birds.

The implication of the restriction to sauropsids is that for that group, *and that group alone*, maximum growth-rate allometry is directly tied to metabolism in the sense discussed above that there is a distinct separation between endotherms and ectotherms. That is directly implied by the Werner and Griebeler conclusion that they can determine the growth rate of dinosaurs, but that you can’t do this more broadly. So in order to support this they must both explain why this *is* the case for sauropsids, and why it *is not* the case when all other vertebrate groups are included in the analysis.

There is no other discussion in [3] to explain or justify why the “restricted to sauropsids” case is valid. Myhrvold [2], rejected this approach for lack of either evidence or theoretical justification and because it cannot be supported on phylogenetic grounds.

The restriction to sauropsids implies that there is a direct link between maximum growth-rate allometry and metabolism for sauropsids in the sense above, and that sauropsids are different than all other extant vertebrates in this regard. In particular, it implies a lower limit to maximum growth-rate allometry for sauropsid endotherms; the maximum growth rate *G*_max_, when adjusted for body size ${\hat{G}}_{max}$ (what Werner and Griebeler term “residual variation—see Eq (3) below and surrounding discussion) cannot be less than a certain value ${\hat{G}}_{max,endo}$. It also implies that there is a maximum upper limit to the maximum growth-rate allometry for sauropsid ectotherms; the maximum growth rate (adjusted for body size) cannot be more than a certain value ${\hat{G}}_{max,ecto}$, and that ${\hat{G}}_{max,ecto}<{\hat{G}}_{max,endo}$.

Werner and Griebeler [3] claimed incorrectly that empirical evidence from the data set that they analyzed on maximum growth rates adjusted for body size shows no overlap between sauropsid endotherms and ectotherms. In fact, as shown in a section below, the groups do overlap. Moreover, an empirical finding of no overlap in a small data set is insufficient absent additional evidence that some heritable trait in sauropsid physiology will enforce the limits ${\hat{G}}_{max,ecto},{\hat{G}}_{max,endo}$. The conclusion that dinosaurs are ectotherms implicitly assumes a biological mechanism: i.e., that dinosaurs inherited a physiological restriction that would make it *impossible* for them to be endothermic with a maximum growth rate lower that the extant birds studied in [3].

Mammalian endotherms do exhibit lower maximum growth rates than extant birds. Conversely, reptilian and fish ectotherms exhibit maximum growth rates higher than many mammals. It is thus clear that this physiological limit is not universal across vertebrates. In other words, it must be unique to sauropsids in order for the “restriction” to sauropsids to apply.

The section of the formal comment titled “Groups used as references for dinosaurs” offers several previously unpublished arguments to support this conjecture [1]:

1. “Extant birds and reptiles are phylogenetically closest to extinct dinosaurs, and birds are even living dinosaurs.”
2. “Since endothermy evolved within the clade Dinosauria [16], extant reptiles are models for ectothermic dinosaurs (ancestral state).”
3. “Birds are models for endothermic dinosaurs”.
4. “Endothermy arose independently in the synapsid and therapsid lineage.”
5. “This is still reflected in considerable differences in energy production and utilization between extant birds and mammals [16].”

It is undisputed that birds are endotherms and a sub-clade of dinosaur (point 3). Extant birds thus must be a component of any comparative study of dinosaurs. There is no requirement that they be the only point of comparison or the only endothermic reference model, however. Birds also lack some features found in dinosaurs: birds do not attain the body sizes of most known dinosaurs from the Mesozoic, for example. Extant birds are unlikely to be a good physiological model on issues relating to body size for a 100,000 kg sauropod.

The evolution of endothermy (points 2 and 4) is a controversial and much-debated topic. The abstract of one recent paper on this topic begins: "Endothermy and its evolution are still an unresolved issue”; the authors are Werner and Griebeler [17]. New discoveries about extant groups abound, including fully endothermic fish [18], seasonally endothermic reptiles [19], and a seasonally ectothermic mammal [20–22]. New insights into extant groups also come from novel molecular and physiological approaches [23–31]. Anatomical features that have long been associated with endothermy, like respiratory turbinates, have been questioned [32]. In addition to new lines of evidence, new hypotheses have been suggested for reasoning about the evolution of endothermy [17,21,22,24–26,28–30,33–38].

With respect to the evolution of endothermy within Dinosauria, several possibilities were discussed in Myhrvold [2] and illustrated graphically in its Fig 1. One possibility is point 2 above that endothermy evolved within Dinosauria, but if so, we don’t know when, or where within the tree. There is some evidence of a physiological change within the Therapoda on the lineage leading to birds [33], but the evidence hardly dispositive. Even if there was a difference then, it is unclear that the difference was endothermy—it could have instead been one form of endothermy to another.

There is no reason to believe a priori that the 17 taxa studied by Werner and Griebeler [3] all must have the same metabolic rate—they span a diverse phylogenetic tree and differ in age by ~145 million years [2]. It is possible that some were ectotherms, but it is also possible that some are endotherms [39].

Alternatively, endothermy might have evolved prior to any of these specific taxa [40] and been ancestral to dinosaurs, contra point 2. While reptiles are the next closest extant group after birds, the pterosaurs are more closely related to dinosaurs than extant reptiles. Most studies conclude that pterosaurs were likely endotherms [41]. Other independent lines of evidence suggest that high metabolic rates could be ancestral to all archosaurs [42], and that crocodiles may be secondarily ectothermic [43]. Middle Triassic archosaurs appear to have had fast growth rates [44]. Evidence suggests that at least some early therapsids had elevated body temperature [45]. These new arguments must be brought into the debate about the evolution of endothermy.

The authors support point 5 with a reference to a 2010 study [46] that presents a hypothesis on the evolution of endothermy based on advances in understanding ATP generation by mitochondria. Since that time, more recent advances have entered the debate, as referenced above. Subsequent papers by the lead author of [46] show that the pattern of endothermy between birds and mammals shares some features while differing in others [39,47], and that across the clade Dinosauria there may have been a diversity of both metabolic rates and thermoregulation strategies [39].

Taken together, points 2 through 5 are, at best, arguments as to why extant reptiles and birds are two reasonable reference models for comparing to dinosaurs. But the developments noted above make it clear that other reference models are also possible.

The physiological argument advanced in the comment must explain both (a) why reptiles and birds both have specific physiological features that link their growth rates and metabolism, and (b) why those same features are entirely absent in mammals and fish. Without both (a) and (b), it cannot be physiologically valid to restrict the comparison as the authors seek to do. Yet neither the formal comment [1] nor any of the papers referenced in it supply these necessary explanations.

Point 1 makes a phylogenetic argument. A simplified phylogeny of the groups within amniotes relevant to this argument is shown in Fig 1. In addition, we cannot ignore the lack of evidence that a link between metabolism and growth rates works when taken across all extant groups.

The phylogenetic argument is fallacious, as can be shown by any of the multiple ways listed below. (For simplicity, the discussion here uses the same groups as Werner and Griebeler.)

**Fig 3:**
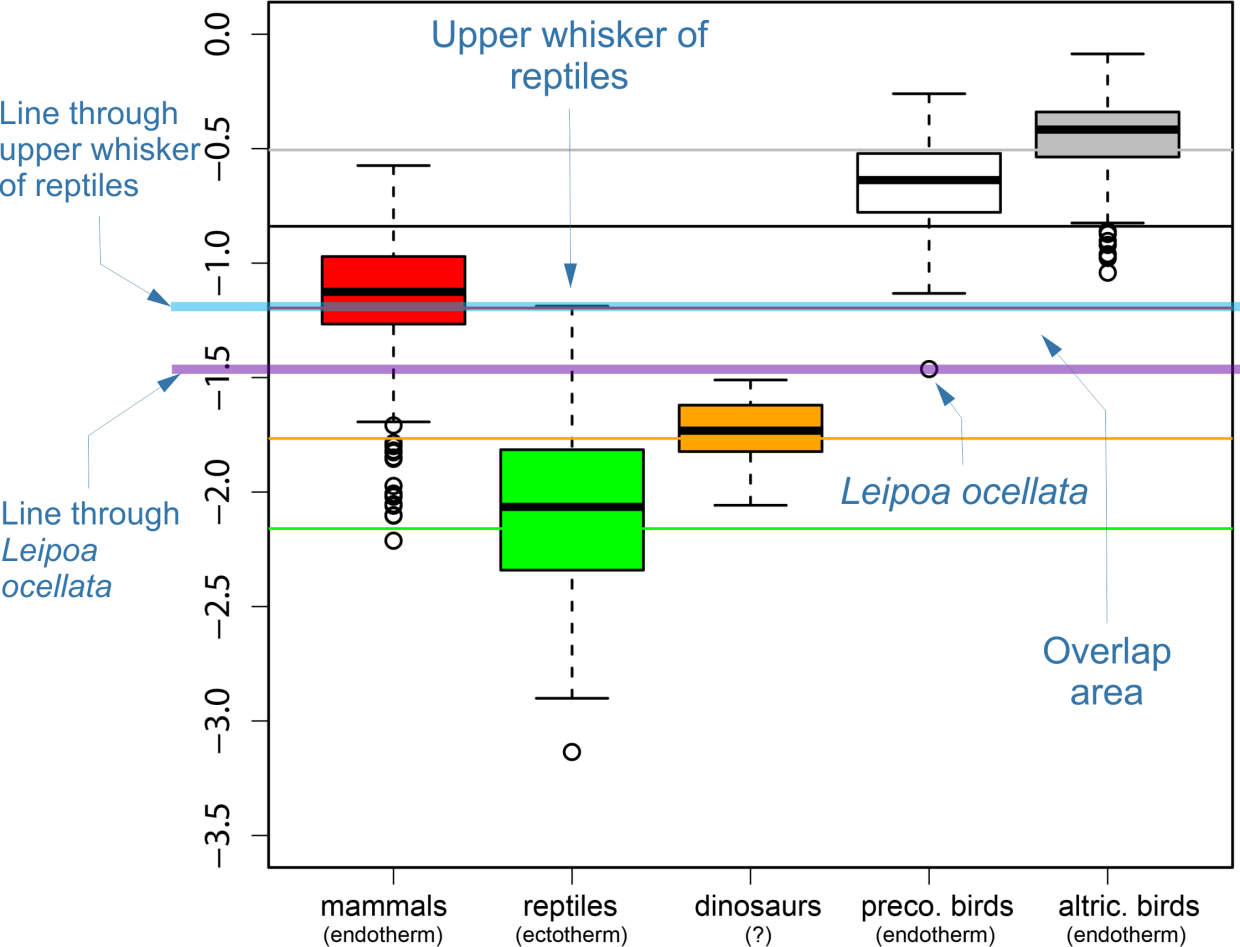
Demonstration that extant sauropsids endotherm and ectotherm growth rates overlap in the Werner and Griebeler study. The upper portion (i.e. minus the phylogenetic tree) of Fig 2 of Werner and Griebeler [3] is reproduced with text annotations in blue, and with transparent horizontal blue and purple lines added. This figure is a box and whisker chart of the distribution of residual variations see Eq (3) for each group. The transparent horizontal blue line passes through the upper whisker of the reptile residuals. The transparent purple horizontal line passes through the lowest data point in the precocial birds, which belongs to *Leipoa ocellata*. Since the purple line is *below* the blue line, the precocial bird residual variations (Eq (3)) overlap with the reptile residual variations, contrary to the statements in Werner and Griebeler [3] and the formal comment [1].

1 The Sauropsida is the name of an interior node on the phylogenetic tree for vertebrates. Such nodes are not, by themselves, hard borders that must not be crossed by phylogenetic comparative biology. These interior nodes have no special status other than representing the legacy of common ancestry. Simply because dinosaurs are classified within Sauropsida is not a valid reason to avoid comparisons with other groups. Indeed, if it were invalid to draw comparisons outside a clade at this level, then much of comparative biology would be forbidden. Mammals are the only extant synapsids, a group that is a peer to sauropsids. So we could not compare them to the anything else. Within what Werner and Griebeler term “fish” we could not compare the teleosts to the chondricthians, much less compare either of those with mammals, reptiles or birds.
2 Birds are contained within the clade Dinosauria, so from a phylogenetic standpoint birds *are* dinosaurs. The most parsimonious (i.e. fewest homoplasy) assumption is that birds inherited the metabolic status of dinosaurs on their lineage. To confirm this one would lump Dinosauria together (i.e. birds+dinosaurs from Werner and Griebeler) and compare to the most closely related out-groups. The out-groups to compare the bird+dinosaur group to are mammals and reptiles. Since mammals and reptiles overlap, we conclude that we cannot determine the metabolism because birds+dinosaurs growth rates combined overlap with both reptiles and mammals.
3 Note that the “bird” groups for Werner and Griebeler [3] do not contain *Archaeopteryx*, which is unaccountably included with dinosaurs. As discussed in Myhrvold [2], the growth rate data for this taxon point is essentially a guess and likely should not be used at all. However, if one is going to use it, and if one is going to separate birds from dinosaurs, then why would *Archaeopteryx* be put with dinosaurs? There are two alternatives–one is to put all of the birds with dinosaurs (i.e. 1. above), while the other is to put *Archaeopteryx* with birds. If that is done, then the birds and reptiles overlap and we cannot determine dinosaur metabolism.
4 Phylogeny is about relatedness, and while it is true that birds and reptiles are the most closely related groups, there is no principle in phylogeny that one must only compare to nearest neighbors on the phylogenetic tree. Indeed when a trait is shared across many groups (as both endothermy and ectothermy are) one is *compelled* to look at it broadly. As a case in point, we cannot dismiss the fact that the growth rate–metabolism link fails when attempted across extant groups.
5 Comparative biology is not limited to phylogenic based comparisons of closest relatives. The study of convergent evolution instead uses ecological or physiological factors as the basis for comparison–for example comparing adaptations of aquatic animals, or top predators, or inhabitants of extreme ecosystems, animals which hibernate, etc. Dinosaurs are large bodied terrestrial animals, and it would be entirely valid and appropriate to compare to them to other large terrestrial animals. Birds all either fly, or are secondarily flightless. They also have much smaller body size than dinosaurs (by any statistical measure–minimum, maximum, mean, mode …) both of which could be argued to make them less suitable as a point of comparison than mammals.
6 The Extant Phylogenetic Bracket (EPB) is a method that is used to infer the properties of extinct groups [16] by careful comparison with features of the most closely related extant groups. In the formal comment [1] it is argued that one cannot use birds as an outgroup for EPB because “Birds are living dinosaurs and thus they are no living outgroup”. This is mistaken. The EPB was specifically developed to understand features of dinosaurs, using birds as part of the extant bracket [16]. A recent query of Google Scholar shows more than 500 papers using EPB for dinosaurs using birds as an outgroup. Nevertheless, if we were to accept their extremely unconventional view that birds cannot be used as one component of the extant bracket, then we must turn to the two other most closely related groups that share both endothermy and ectothermy–namely mammals and reptiles. Since they overlap, we conclude we cannot determine dinosaur metabolism. Eliminating birds as an EPB bracket outgroup does not help.
7 If instead we adopt the conventional approach to the EPB and we do use birds and reptiles as the comparison groups. However that does not help either, because we must also check the reptiles against their nearest neighbors—viz., the marsupials and eutherian mammals. This is to verify that reptilian growth rates can, when compared to those of their nearest phylogenetic neighbors, be diagnostic of their ectothermy. That test obviously fails: mammal growth rates overlap with those of reptiles (see Fig 2 of Werner and Griebeler [3], or its excerpt in Figs 2 and 3), yet members of the two groups differ in metabolic status.

8 The question under consideration is whether the link between maximum growth-rate allometry and metabolism is strong enough for sauropsids that we can use it to classify metabolism. As established above, we know that this is *not* true generally–we *cannot* classify extant vertebrate groups broadly by metabolism using growth rate allometry. So in order to do this for sauropsids the following four propositions must *all* be true:
  a There is compelling evidence that the physiology of sauropsids has specific features such that sauropsids have a strong link between maximum growth rate and metabolism; in particular that sauropsid endotherms are prevented from having maximum growth rates (adjusted for body size) that are lower than extant birds (i.e. the limits ${\hat{G}}_{\max,\mathrm{ecto}}$, ${\hat{G}}_{max,endo}$ discussed above.)
  b These features must be *unique* to sauropsids. Without this uniqueness the fact that the link fails in other groups (synapsids and teleosts) would void its applicability. In other phylogenetic terms, a metabolism growth-rate link must be a synapomorphy for Sauropsida and a plesiomorphy for synapsids and teleosts.
  c The maximum growth rates (adjusted for body size) must not overlap between sauropsid endotherms and ectotherms.

The Werner and Griebeler study [3] makes point (c) above (but see below), however it does not offer any suggestion of evidence for (a) or (b). Indeed I am not aware of any suggestions to that effect. Without strong evidence of (a) and (b) we must reject this option because it would imply that maximum growth rate and metabolism is linked in sauropsids, but not in the groups most closely related to them.

9 Werner and Griebeler [3] followed Case in treating altricial and precocial birds separately (see the discussion of group composition in Section 8). This shows that multiple separate lineages of birds evolved two different strategies for ontogeny [49,50], which allowed them to span different ranges of growth rates. In the Werner and Griebeler framework, dinosaurs must be ectotherms because their growth rates are (claimed to be) below the minimum threshold for sauropsids endotherms. But sauropsids endothermy is clearly flexible enough to allow at least two different strategies, so why would it not be flexible enough to allow dinosaurs to evolve their own strategies? That could include some that would allow endothermy with lower growth rates (as demonstrated by mammals).
10 Several studies have suggested that large body size may have led to novel thermoregulatory approach for dinosaurs, usually called inertial homeothermy or gigantothermy [24,39,46,51]. Researchers have proposed other novel metabolic or thermoregulatory approaches [39]. This is also mentioned as a possibility in the GW comment. To the extent that this is a possibility, we cannot compare only to extant birds, as they are too small for this effect to be relevant. The extant groups that have some degree or another of gigantothermy in some species are reptiles [51–54], mammals [55], and fish [56,57]. Since the 17 dinosaur taxa studied in Werner and Griebeler [3] include sauropods, this suggests that the best comparison to them, and perhaps to some of the other large bodied taxa, would be to these examples.
11 More generally, if we assume that dinosaurs had truly novel mechanisms for metabolism or thermoregulation (or both), or a novel connection between metabolism and maximum growth rate not shared with extant vertebrates, then we cannot expect a comparison to extant animals to empower us to deduce their metabolism.

Doubtless other possible arguments exist, including combinations of the ones discussed above. In my view these arguments, taken together with the counterarguments to points 2–5, rule out the validity of restricting the comparison to sauropsids.

## 4. PGLS versus OLS

A critique raised by the GW comment is that Myhrvold [2] used OLS regression, whereas Werner and Griebeler [3] used PGLS. In general, OLS assumes that its data points are uncorrelated, which is false for taxa that are closely related. When a good phylogeny is available, PGLS will compensate for a lack of independence by calculating the degree of relatedness or phylogenetic signal.

The reason that Myhrvold [2] did not use a phylogenetically informed regression method is that one of the fundamental messages of the study is that regression—of any kind—is not a suitable classifier for metabolism, as further discussed in Section 5. That paper demonstrated that the overlap between growth rates that makes classification impossible can be determined without the use of any regression method [2]. Some prior studies sought to use a quantitative comparison of regression parameters in the maximum growth-rate allometry to classify groups as endothermic or exothermic—a hypothesis that Myhrvold [2] referred to as H1 and showed to be false.

Werner and Griebeler [3] did not use regression to classify in this way (see Section 5). Instead they used the “residual variation” after linear PGLS or OLS regression on log-transformed data with a fixed slope of 0.75:

Therefore we used the gnls function [34] in R and the model log10 AGR ~ intercept+log10 BMatMG * 0.*75*.

The regression was therefore determining a single parameter; the intercept. Given the model described in the quote the residuals would be calculated as follows

$$residual=\log_{10}\left(AGR\right)-intercept-{log}_{10}(BMatMG)*0.75,$$ (1)

where *BMatMG* is the body mass at maximum growth rate, *AGR* is the absolute growth rate both of which are in the data set on a per-taxon basis. The intercept is computed by regression on a group (i.e. precocial birds etc.). This was then used to assess the residual variation as follows [3]:

Finally, as a measure of variability in residuals and the deviation of single species from the expected average, we calculated for each regression line the residual variation as residuals+average (intercept) and compared these values between regression models (see also Results).

This can be expressed as the following equation,

residualvariation=rv=residual+intercept. (2)

Werner and Griebeler use the residual variation to assess whether there is overlap between different groups. This is shown in their Figure 2 [3], which is presented here in annotated form as Fig 2 of this response (see discussion below for details of annotations). Note that we can combine Eqs (1) and (2) to yield

$$rv=\log_{10}\left(AGR\right)-{log}_{10}(BMatMG)*0.75={log}_{10}(AGR*{BMatMG}^{-0.75}).$$ (3)

The importance of Eq (3) is that the regression intercept cancels out, leaving the result that the residual variation analysis of Werner and Griebeler is entirely independent of the regression method used. There is no point in computing the residual variation separately for OLS and PGLS, as is done in Werner and Griebeler [3]. That fact renders moot the arguments in the formal comment [1] concerning my use of OLS versus PGLS.

A comparison of the plot of PGLS residual variations (Figure 2 in [3]) to the OLS residual variations (Figure S1 in [3]) shows that they are almost identical–they are plotted side by side in Fig 2 here. They are completely identical with respect to the most important point–the overlap of growth rates (residual variation) between reptiles and precocial birds discussed in the next section. Slight differences in the box and whisker plots occur only because slightly different data sets were used to compute the residual variations. The PGLS data set was limited to taxa which had phylogenetic information, while the OLS plot includes some for which that was not available.

Note that PGLS and OLS certainly do not always yield the same results. They give equivalent results here primarily because the procedure for determining residual variation explicitly cancels out the impact of the regression method used. Put another way, Werner and Griebeler do not actually use regression to determine the overlap between groups. Instead their residual variation is effectively a mass-adjusted growth rate, as shown in Eq (3). A mathematically similar approach was taken by Grady et al., as discussed in Myhrvold [2].

**Fig 4:**
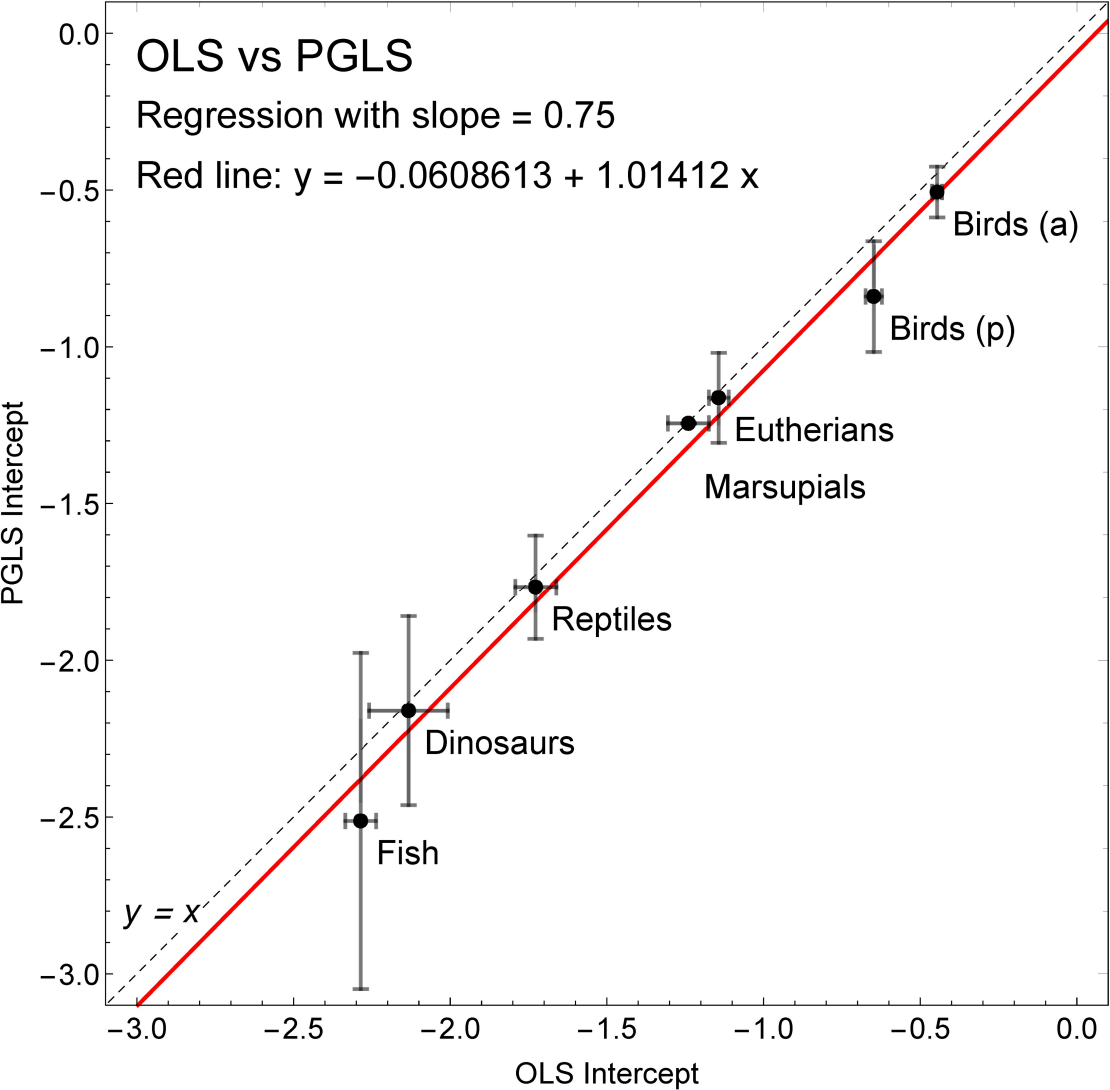
Regression intercepts for PGLS and OLS regression with fixed slope 0.75 from Werner and Griebeler [3]. The values of the regression intercept for PGLS and OLS regression are given in Tables 2 and S3 in [3]. Here they are plotted against one another to show their relationship; error bars show the 95% confidence intervals. If the two methods gave the identical intercept they would like on the line *y* = *x* (*black*, *dashed*), and this lies within the PGLS 95% confidence interval for every group except precocial birds (which are close). The red line is the result of a simple linear fit, with parameters given in the legend. It shows that taken across all groups the primary difference between PGLS and OLS coefficients is a constant offset.

Fig 4 plots the regression intercepts from PGLS and OLS regression on fixed slope 0.75, as derived by Werner and Griebeler and presented in Tables 2 and S3 of [3]. The red line in Fig 4 is a simple linear fit of the OLS vs. PGLS intercepts. It shows that overall the two differ by an offset of -0.061, with a slope very near unity (coefficient of x is 1.01). Every group includes the line *y* = *x* (*dashed black line* in Fig 4) in the PGLS 95% confidence interval except precocial birds (and they are very close)–which means that to the 95% level we cannot reject the hypothesis that PGLS and OLS are *the same for those groups*. This is remarkable given that the two data sets are slightly different (more taxa in the OLS groups than in the PGLS groups).

Since the regression methods cancel out in the Werner and Griebeler analysis (see section 3 above) this does not matter to the final outcome of [3]. However it shows that even if the outcome *did* depend on regression parameter there may not be as much difference as might be expected between OLS and PGLS; a further rebuttal of the criticism regarding PGLS versus OLS raised in [1].

## 5. Sauropsid endotherm and ectotherm growth rates overlap

An independent set of objections to restricting the comparison only to Sauropsida arises if, for the sake of argument, we accept that choice as a counterfactual and examine it closely. In that case, we can ask whether the growth rates on extant sauropsids do actually overlap.

Werner and Griebeler claim they do not, and that assertion is central to their conclusion that they can determine the metabolism of dinosaurs, and that they are ectotherms. If there were overlap it would overturn this conclusion, as the formal comment [1] concedes:

larger samples on endothermic and ectothermic Sauropsida could potentially demonstrate an overlap between reptilian and avian G_max_ values (third aim), which would reject our conclusion that dinosaurs had maximum growth rates as seen in similar-sized (if necessary scaled-up) fast-growing extant ectothermic reptiles.

Careful examination of Werner and Griebeler [3] reveals that the authors’ own work demonstrates that growth rates of ectothermic and endothermic sauropsids do in fact overlap. As illustrated here in Fig 3, an excerpt from their Fig 2 clearly shows overlap between the growth-rate allometry of precocial birds and reptiles.

The precocial bird data point that causes the overlap is due to the malleefowl (*Leipoa ocellata*); the reptiles that cause the overlap are *Aspidoscelis tigris* and *Nerodia erythrogaster*. Both the original analysis by Werner and Griebeler and my own previous examination of their paper during the preparation of Myhrvold [2] overlooked this overlap, but it is indisputably there.

The data on precocial birds used by Grady et al. has different species that that used by Werner and Griebeler, so the two are largely complementary. As a comparison, the largest precocial bird in the Werner and Griebeler data has *BMatMG* of 4.359 kg, whereas in Grady et al. the largest *BMatMG* is more than 8.5 times as large, at 37.376 kg, because that data set include all of the large ratites (cassowary, emu, rhea, ostrich).

When the data of Grady et al. is adjusted for *BMatMG* (multiplying body mass by 1/*e*) so as to be compatible with the Werner/Griebeler data sets (see section below), the extent to which precocial birds overlap with reptiles is even greater. Pooled together, there are four bird species, and five reptiles in the overlap zone. The numerical data for these overlapping species are shown in Table 1. The numerical values demonstrate the overlap and are easy to verify since the calculation is so simple (i.e. via Eq (3)).

**Table 1:** Sauropsid species with overlapping residual variation. The sauropsids species which have overlapping residual variation (in the sense of Werner and Griebeler [3] and Eq 3)) are shown. The raw data for each species (from supplementary online information of [3]) is body mass at maximum growth (*BMatMG*) in grams and the maximum growth rate *AGR* (also called *G*_max_ in [2] and formal comment [1]) in grams per day, as found in the references in the rightmost column. The column *AGR* * *BMatMG*^−0.75^ is 10^*rv*^ for residual variation *rv* given by Eq (3). The table is sorted in descending order by *rv*. The metabolic status (endotherm or ectotherm) of each species is shown and highlighted (endotherms in *red*, ectotherms in *blue*). In the Ref column, WG refers to of Werner and Griebeler [3], Grady to Grady *et al*. [14].

Species	Common Name	*BMatMG*	*AGR*	*AGR* * *BMatMG*^−0.75^	Metabolism	Ref
*Aspidoscelis tigris*	Western whiptail lizard	1.778	0.1	0.0649	Ectotherm	WG
*Phasianus colchicus*	Common Pheasant	436.673	6.05	0.0633	Endotherm	Grady
*Pavo cristatus*	Peacock	1265.14	10.38	0.0489	Endotherm	Grady
*Nerodia erythrogaster*	Plainbelly water snake	47.407	0.86	0.0476	Ectotherm	WG
*Agama impalearis*	Bibron’s agama lizard	22.0728	0.39	0.0383	Ectotherm	Grady
*Leipoa ocellata*	Malleefowl	587.136	4.11	0.0345	Endotherm	WG
*Phrynosoma solare*	Regal horned lizard	14.815	0.25	0.0331	Ectotherm	WG
*Uta stansburiana*	Side-blotched lizard	1.481	0.04	0.0298	Ectotherm	WG
*Apteryx mantelli*	Brown Kiwi	792.412	4.291	0.0287	Endotherm	Grady

A potential explanation is to regard the point for *Leipoa ocellata* as an outlier to be dismissed. It is plotted as such in Figure 2 in [3], but only because of the defaults present in the R language whisker plot routine (as explained in the caption to Figure 2 in [3], which does the same to data points in several other groups.) Formal classification of taxa as outliers is inappropriate with a simple rule such as the R whisker plot default (>1.5 times the interquartile distance), because variations in growth rates and body mass are fundamentally biological parameters, *not* results of randomness in stochastic models. Discarding data points, even those considered “outliers”, would be unethical without both justification and disclosure [58].

**Fig 5:**
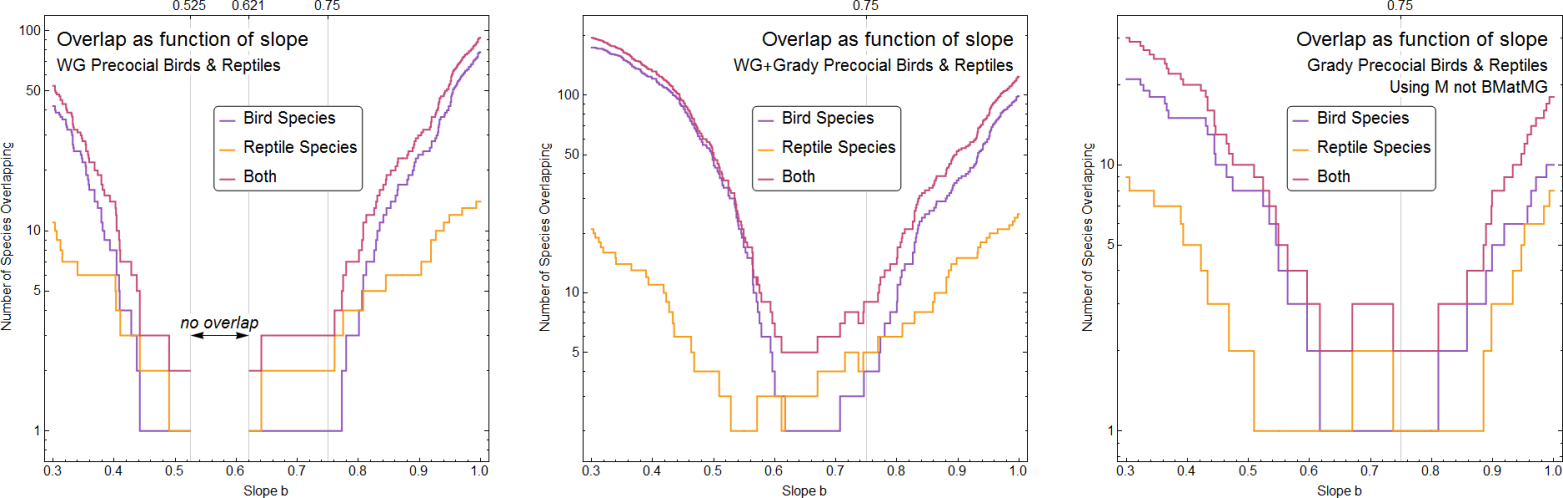
Counts of residual variation overlap as a function of slope *b*. The count of species in the reptile and precocial bird with overlapping residual variation is shown for values of slope *b* other than the value *b* = 0.75 used by Werner and Griebeler [3]. For the data set used in [3] (*left*) there is overlap for 0.525 ≤ *b* ≤ 0.621, but other values of *b* do have overlap. When the Grady et al. [14] data set is included (*center*), there are species in the overlap region for all values of *b*, and this remains true for the data by itself, using *M* rather than *BMatMG* as the mass variable (*right*).

The residual variation analysis above uses *b* = 0.75 in Eq (3) to make a mass-adjusted maximum growth rate. The phenomenon of overlap between precocial bird and reptile mass-adjusted growth rates (or equivalently, residual variation in [3]) is robust to other values of *b*. This is shown in Fig 5, which plots the count of overlapping species between reptiles and precocial birds. The Werner and Griebeler data set [3] shows no overlap for 0.525 ≤ *b* ≤ 0.621, but all other values of *b* do have overlap. This is important because *b* = 0.67 is expected from surface to volume ratio, and many empirical studies support *b* > 0.621 but *b* ≠ 0.75 [59–67].

When the Grady et al. data set [14] is included there are species in the overlap region for *all* values of *b*, and this remains true for the Grady et al. data by itself, using *M* rather than *BMatMG* as the mass variable (see section 9 below). The choice of slope *b* is thus not a major factor in overlapping growth rates between reptiles and precocial birds; the phenomenon is quite robust.

## 6. Hypotheses H1 and H2

The formal comment [1] raises a number of additional technical points regarding Myhrvold [2] that have little or no bearing on the ultimate conclusions about whether growth-rate allometry is linked to metabolism or the metabolic status of dinosaurs. One of these concerns the discussion in Myhrvold [2] of its hypotheses H1 and H2 and how that relates to the characterization of Werner and Griebeler [3] in [2]. Nevertheless, since the formal comment [1] takes issue with these points quite vigorously with much text devoted to them, they warrant a response.

In Myhrvold [2] it was important to explicitly and clearly state the proposition about growth rates and metabolism that had been used in the series of dinosaur growth rate studies [6–13], and Grady *et al*. [14], particularly since the proposition had not been clearly stated, and was often implicit in those studies. So two hypotheses, H1 and H2 were formulated to capture the differing ways that these authors linked metabolism and growth rate.

H1 is stated in Table 2; it involves using regression parameters. As discussed above the primary conclusion of the Werner and Griebeler study [3] that one cannot determine metabolism in extant groups from maximum growth-rate allometry is *not* an example of H1, at least in practice because Eq (3) demonstrate that Werner and Griebeler do not use regression at all in determining overlap between groups.

**Table 2:** Quotations relevant to hypothesis H1 in [2] and [3]. Passages from Werner and Griebeler [3] that express a view equivalent to hypothesis H1 are shown *above*, along with passages from Myhrvold [2] defining H1, and reaching a conclusion about Werner and Griebeler [3] with respect to H1.

**Quotes from Werner & Griebeler [3]**	
Page 7	What can we infer from growth rates of dinosaurs about their metabolism or thermoregulation strategy? Our results suggest that dinosaurian growth rates were between those predicted by the reptile and mammal regression model and that some dinosaurian growth rates were within the range of mammalian growth rates. Thus, one may argue that at least some dinosaurs were endotherms and/or had metabolic rates similar to those of mammals.
	Additionally, nonavian dinosaurian PGLS regression coefficients (intercept and slope) did not differ statistically from coefficients of the reptile regression model. All these arguments provide evidence that the studied dinosaurs had a lower metabolic rate than recent endotherms.
**Quotes from Myhrvold [2]**	
Page 2	H1. The metabolism of all members of a taxonomic group is determined by the regression parameters *a* and *b* for the group (from the allometric relationship *G*_max_ = *a M*^*b*^), by comparison with *a*, *b* for groups with known metabolism.
Page 6	Werner and Griebeler [12] recognized at least some of the problems with hypothesis H1.

One of the problems that Myhrvold [2] found with H1 is that it is an example of the “ecological fallacy”, which is a well-known problem in statistical reasoning. The essence of this point was clearly understood by Werner and Griebeler who wrote [3]:

While the averages (regression lines) indicate a clear separation, individual growth rates overlapped between several taxa, even between endotherms and ectotherms (Figs 1 and 2). This indicates that an assignment of a species to ectotherms or endotherms solely based on its growth rate is not possible and that it is inappropriate to apply Case’s or our allometries at the single species level.

A portion of this passage was quoted approvingly in Myhrvold [2], and the overall conclusion regarding Werner and Griebeler’s use of H1 is entirely *positive*, as shown in Table 2.

Note however that there are two passages in Werner and Griebeler [3] that do invoke H1; these are shown in Table 2. These passages are taken from the discussion of arguments presented in support of two alternatives–the “unrestricted” case where comparison to mammals is allowed, and the “restricted” case where comparison is limited to sauropsids (see sections above). Each passage describes comparing the dinosaur regression coefficients (i.e. *a*, *b* from *G*_max_ = *a M*^*b*^) from the H1 definition above and comparing them to the reptile regression coefficients as evidence that the dinosaurs are ectotherms. This is a clear statement of H1, and it is used to “provide evidence” in support of the main conclusion of [3] that dinosaurs were ectotherms (i.e. the analysis which restricts the analysis to sauropsids).

Thus the summary of the treatment of Werner and Griebeler [3] with respect to H1 is Myhrvold [2] overlooked the fact that they invoked an H1 argument as a secondary supporting factor for their main conclusions. The arguments in the formal comment [1] disputing their involvement with H1 are thus misplaced. Myhrvold [2] never claimed that Werner and Griebeler [3] used H1; although it easily could have done so because [1] does invoke H1 in a subsidiary way.

The situation with H2 is similarly indirect. H2 is primarily proposed in Grady et al. [14], and is stated in Table 3.

**Table 3:** Quotations relevant to hypothesis H2 in [2] and [3]. Passages from from Werner and Griebeler [3] that express a view equivalent to hypothesis H2 are shown *above*, along with passages from Myhrvold [2] defining H1, and reaching a conclusion about Werner and Griebeler [3] with respect to H2.

**Quotes from Werner & Griebeler [3]**	
Page 7	The same order of intercepts of the studied taxonomic groups is observed for metabolic rates [37,49,59,60] and growth rates. Additionally, similar slopes are observed in regression models of growth rate against body mass or metabolic rate against body mass [5,9,10,12,14,20,37,49,59–61]. Thus a link between growth rate and metabolic rate seems very likely. Consequently, the differences observed in growth rates might be caused by the different metabolic rates of the taxa.
	Nevertheless, very high growth rates seem to be linked to a high (basal/resting) metabolic rate and a considerable level of parental care in extant organisms.
**Quotes from Myhrvold [2]**	
Page 2	H2. The basal metabolic rate (*BMR*) is directly related to maximum growth rate *G*_max_ by an allometric equation *BMR* = *αG*_max_^*β*^ for constants *α* and *β*.
Page 6	I examine below each of the statistical and biological arguments used by the Werner and Griebeler and Grady et al. studies in favor of H1 and H2.
Page 23	Werner and Griebeler similarly noted that “Additionally, similar slopes are observed in regression models of growth rate against body mass or metabolic rate against body mass [5,9,10,12,14,20,37,49,59–61]. Thus a link between growth rate and metabolic rate seems very likely” [12].

The top quotation from Werner and Griebeler in Table 3 is simply a plain-language statement of the hypothesis H2 equation. The regression of metabolic rate against body mass is the central equation of MTE:

$$MR=B_{0}(T)\;M^{0.75},$$ (4)

where *M* is the body mass *MR* is metabolic rate and *B*_0_ is a normalization term that depends on body temperature *T* (see discussion below) [68,69]. MTE also predicts that the slope *b* in the H1 equation is *b* = 0.75, and that is the value that Werner and Griebeler use. The link between growth rate and metabolic rate that they say “seems very likely” is the H2 equation *MR* = *αG*_max_^*β*^.

The idea that *G*_max_ ∝ *M*^*b*^, and *MR* ∝ *M*^0.75^ implies *MR* ∝ *G*_max_^*β*^ may seem quite natural. Indeed, if each relationship was based on pure mathematical equality, it would be true for *β* = 0.75/*b*. This is what is expressed in the passage above by: “Additionally, similar slopes are observed… Thus a link between growth rate and metabolic rate seems very likely”. However, in reality each of the relationships *is a statistical correlation*, and these are *not transitive*. This is a well-known statistical fallacy that is discussed at length in Myhrvold [2]. It is quite distinct from the ecological fallacy.

The passage from Werner and Griebeler [3] which states H2 occurs in a section discussing various alternatives. It ends with the second passage quoted in Table 3 that “Nevertheless”, growth rate and metabolism seem to be linked. This statement of H2 seemed sufficiently strong that Myhrvold [2] including Werner and Griebeler as being “in favor” of H2 (comment on page 6 in Table 3). It also said that [3] “noted” that the link between the two was likely.

In light of the passages in Table 3, Griebeler and Werner should not “wonder”, as stated in their formal comment why this is mentioned in my paper [2]. It was entirely fair to quote them as “noting” that they thought a link was likely.

The situation with H1 and H2 is thus very similar. Neither is central to the primary analysis that Werner and Griebeler present; their main thrust is elsewhere. However, the written text of the paper *does* mention both hypotheses H1 and H2 as supporting arguments, and as a result I think that it is fair that I commented on them in [2].

## 7. The use of metabolic theory of ecology in Werner and Griebeler [3]

The next issue does not have any direct impact on conclusions, but it is treated at length in the formal comment [1] and was contentious during review of this response. Whereas Grady et al. [14] relied heavily on the metabolic theory of ecology (MTE), creating issues raised in [2], Werner and Griebeler [3] depended far less on MTE, but did have an apparent dependence which was also raised in [2]. This is viewed as problematic in the formal comment, and in review feedback on an earlier version of this response.

In order to facilitate evaluation of this issue, Table 4 contains all quotes in [3] mentioning MTE, or to “theory” or “theoretical” (which in context means MTE), and the relevant quote in [2] regarding the use of MTE in [3].

**Table 4:** Quotations regarding MTE in [2] and [3]. All sentences from Werner and Griebeler [3] containing “MTE” (metabolic theory of ecology) or “theory” or “theoretical” are shown in, along with the quote in Myhrvold [2] characterizing the use of MTE in [3].

**Quotes from Werner & Griebeler [3] regarding MTE**	
Abstract	For all taxonomic groups, the slope of 0.75 expected from the Metabolic Theory of Ecology was statistically supported.
Page 2	First, we calculated PGLS regressions on log-log-transformed data and tested if the slopes of our regressions were consistent with the theoretically assumed value of 0.75 (AGR should scale as metabolic rate with 0.75 [5,20].)
	In general, PGLS slopes of all studied taxa coincide well with the value of 0.75 predicted by theory (also OLS slopes see Table S2). With the exception of altricial birds and fishes, the PGLS slopes of the studied groups did not differ statistically from 0.75
Page 4	Lines shown are regression lines with a slope forced to 0.75 (consistent with our empirical findings and with theory).
	Comparing Taxa using the Theoretically Predicted Value of 0.75 as the Fixed Parameter in the PGLS Regression Model (section heading)
Page 6	Thus, our results corroborate the Metabolic Theory of Ecology (MTE), which predicts a slope of 0.75 for the allometry on maximum growth rate and body mass [5,20].
	However, it is beyond the scope of this paper to test the assumptions and predictions of the MTE or any other theoretical model on scaling of life history traits with body mass. Whatever the underlying mechanisms are, the accordance of our empirical findings with theoretical predictions and other empirical findings (e.g. [5,20,45–49]) strengthens our approach: using a slope of 0.75 as the fixed parameter in our regression models
**Quote from Myhrvold [2] characterizing MTE use in [3]**	
Page 22	Both Grady et al. [13] and Werner and Griebeler [12] offer theoretical explanations based on MST, MTE, and the body of work that has risen around the allometric scaling of *BMR* with mass first discussed by Kleiber in 1932 [42,43]

Table 3 clearly shows that [3] paper uses MTE as a theoretical justification for a key method in their paper (the 0.75 slope or scaling exponent). They also list corroboration of MTE as a result of the research important enough to mention in the abstract. This corroboration is also a supporting factor that their work is consistent with MTE. The corroboration point accurately described in the second quote on page 2 –two groups do not corroborate MTE (slope *b* ≠ 0.75 in Table 1 of [3]. Yet in the abstract this becomes “all groups”, which overstates the actual finding.

The characterization of MTE use by Werner and Griebeler quoted in Myhrvold [2] also appears in Table 2. In my opinion it seems entirely supported by the quotes above. However, the formal comment [1], argues that in fact Werner and Griebeler [3], “Question the MTE”. In the review on an earlier version of this response the issue was contentious; Dr. Griebeler stated that Werner and Griebeler [3] “make no use” of MTE, and that “The Werner & Griebeler 2014 paper provides in fact no support for the MTE”. I find these statements difficult to reconcile with Table 2.

The formal comment [1] references the following passage in [3] to show that they “question” the MTE:

Metabolic rates of marsupials are 70–90% lower than in eutherians (McNab 1988) and this correlates well with our observed differences in AGRs between marsupials and eutherians (Table 2). However, comparing bird and mammal basal metabolic rates showed that they do not differ more than 1.5 times [35,62,63] on average, whereas AGRs in birds are 2 to 4.5times higher than in mammals. Brown et al. [5] suggested that, excluding body mass, (internal) temperature is the main factor causing the differences in (basal) metabolic rates between similar sized organisms of different taxa. Also, their model predicts only a 1.5 times higher metabolic rate (e2E/(k*Tb_birds)/e2E/(k*Tb_mammals), E = 0.63, k = 8.6173324*1025 (Boltzmann constant), Tb_birds = 314u Kelvin (41uC), Tb_mammals = 309u Kelvin (36uC)) for birds in comparison to mammals. Field metabolic rates vary only 1.2 (100 kg species) to two-fold (1 g species) between birds and mammals [60]. This suggests that metabolic rate alone cannot account for the differences seen between avian and mammalian growth rates.

While it is true that Brown et al. [70] (their reference [5] above) deals with the MTE, this passage does not make any clear argument against the theory, which is not even mentioned here by name. Although the authors do point to inconsistencies between actual metabolic rates and those predicted by body temperature (see discussion below about temperature and MTE), they draw no conclusion from these differences that directly contradicts the MTE. Instead, the conclusion reached is that metabolic rate cannot fully account for the avian vs. mammal growth rates rather than clearly stating that MTE is wrong, although that may be an indirect consequence.

Note that MTE is also a factor in the effect of temperature on metabolism discussed in a section 10.

## 8. The choice of dependent variable: *kC* versus *G*_max_

Another methodological criticism made in the formal comment [1] is the following:

Myhrvold [10] additionally suggested in his paper that the mass-specific growth rate (kC = Gmax/M) should be preferred over the maximum growth rate Gmax (termed maximum absolute growth rate in Werner and Griebeler [8]) when establishing scaling relationships on vertebrate growth rates.

More precisely stated, the question is what dependent variable to use in the regression: the maximum mass-specific growth rate or the maximum absolute growth rate? Myhrvold [2] observed that regression on *G*_max_ can create a false impression of a strong correlation because the data points appear to be tightly clustered, a result of the fact that *G*_max_ has mass *M* as an explicit factor. The false impression of a strong correlation between maximum growth rate and metabolism appears to have been a factor in the incorrect interpretation of the Case results by a series dinosaur growth rate papers [6–13].

However, as clearly pointed out in Myhrvold [2], apart from measures like the coefficient of determination *R*^2^, which assess the absolute scatter in the points, regression on *G*_max_ or *kC* give identical regression coefficients when adjusted for the obvious coordinate transformation. For example if a regression on *G*_max_ yields a regression model *aM*^*b*^, then regression on *kC* will yield *aM*^*b*−1^. The error bounds on *a*, *b* will also be the same.

Because of this mathematical relationship, there is *no difference* in using the two approaches. The mathematics are the same, and thus the impact on biology is the same. Indeed the situation is simply a change in coordinate system, analogous to a change in units. The way this would be expressed in everyday life is that *kC* is the percent growth per unit time, while *G*_max_ is the absolute growth in mass per unit time. It is obvious that both can be used to describe growth, although one must take care not to confuse them.

Yet despite this being clearly articulated, and mathematically straightforward, the formal comment [1] argues that the *kC* approach is somehow deficient and inferior. That is simply not mathematically possible. One scenario described in the formal comment [1] and illustrated in their Fig 1, concerns a series of hypothetical species which have different values of *M* but the same value of *kC*. They find this situation paradoxical, stating:

The latter implies no effect of M on kC, and a scaling exponent of zero. Contrary, when plotting Gmax against M or against BMatMG (body mass at which the maximum growth rate is observed, Werner and Griebeler [8], Fig 1D) for these species we have a unique relation between Gmax and M (BMatMG).

Their paradoxical result is exactly what should happen. If they hypothesize that the identical value *kC* occurs for different values of *M* (or *BMatMG*) then they have created a scenario where *kC* is *independent* of *M* (or *BMatMG*). By definition this implies no correlation between the two and a horizontal regression line. That is built into their chosen scenario. In that situation the only correlation between *G*_max_ and *M* is the trivial correlation caused by *M* being a factor of *G*_max_.

Put another way, one should not complain that the mass-specific maximum growth rate *kC* is, in fact, mass-specific. So when you construct a hypothetical situation where the mass-specific growth rate does not depend on mass, well, then it doesn’t depend on mass. There is nothing strange or paradoxical about that.

There is no particular reason that one has to use the “*kC* approach”. I must emphasize again that it is *mathematically equivalent* to the *G*_max_ approach. The only advantage of *kC* is that it eliminates the trivial correlation caused by the explicit factor of *M* (or *BMatMG*), and thus prevents over confidence in an illusury level of correlation when instead it is an artifact of the chosen dependent variable.

The mathematical equivalence also means that it is unnecessary for me to rebut any other points raised in the formal comment [1] related to the superiority of one approach versus another.

## 9. Choice of independent variable: *BMatMG* versus *M*

Having discussed the dependent variable, I turn now to the independent variable used in regression. Werner and Griebeler use *BMatMG* (body mass at maximum growth rate) as their independent variable rather than the asymptotic mass *M*, their reasoning is given here [3]:

Thus, maximum growth, which is observed at the point of inflection and is expressed in absolute maximum growth rate, is not comparable even between these standard models without an appropriate transformation.

The valid point here is that in different growth models (such as logistic, Gompertz, von Bertalanffy) the inflection point where maximum growth rate occurs is at different values of the body mass for each model. That is a clear and obvious mathematical implication of the models. Werner and Griebeler have decided that makes them “not comparable”. No justification is given in [3] other than the line quoted above.

This is an unusual approach. These models have been extensively used by biologists for decades, and the earliest date to the 19^th^ century. Case [4,5], the dinosaur growth papers referencing Case [6–13] and Grady et al. [14] all use asymptotic mass *M* as the dependent variable. So do countless other allometric studies of metabolism, such as those both for and against MTE (see [2] and references therein).

To my knowledge no other study else has made the inference that maximum growth rates derived from growth model fits are “not comparable”. Any growth data that is well fit by one of these models can also be fit by another, but the fit might not be quite as good. Some studies use multiple models and choose between them by an information theoretic criterion like AICc, while others simply choose a model without any obvious objective reason. The fact that some models fit certain species better than others is well known (see Myhrvold [72] and references therein.) As a consequence, some species have their maximum growth rate occur at different times, and at different body masses, than other species.

In Myhrvold [2] the unusual nature of using *BMatMG* was noted, the passage above was quoted, and after some discussion this was my conclusion:

In effect, Werner and Griebeler present a different biological hypothesis than Case, Erickson, or Grady et al.: viz., that the choice of independent variable matters, and that the choice should be body mass at the age when maximum growth occurs (*BMatMG*). Werner and Griebeler did not discuss their reasoning in detail, nor did they provide a sensitivity analysis assessing the difference this choice makes.

I think that this is correct assessment of the situation. Werner and Griebeler made a choice that differs from other studies. They justify it only with the assertion that they are “not comparable”. I don’t think my conclusion is unfair. It is entirely normal for a scientist to ask for more justification. A sensitivity analysis is also more than warranted to see if this choice would change their results.

My guess is that a sensitivity analysis might not show a qualitative difference to their key conclusions. This guess is based on the fact that the difference between *M* and *BMatMG* is a constant factor *d* which is discussed in both Werner and Griebeler [3] and Myhrvold [2] (in Equation (12)). If all taxa in an analysis have the same sigmoidal growth model, then this simply is shifts the data points over and makes no little qualitative difference in the analysis. If there are three different models, say logistic (*d* = 1/2), Gompertz (*d* = 1/*e*), and von Bertalanffy (*d* = 8/27), then the mass coordinate of the data points would shift by those factors, but only the relative shift matters to cross-group comparisons. Note that the mass coordinate for the vertebrate groups studied by Werner and Griebeler typically span ranges of many powers of 10, so shifting some points by less than a factor of 2 (on a relative basis) from other points would in most cases not make a large quantitative difference and perhaps no qualitative difference.

It would also shift the relative values of the residual variation of Eq (3) a bit, but again would probably not make a large qualitative difference. Fig 5 shows that for the Grady et al. [14] data, using *M* rather than *BMatMG* does not alter the fact that reptiles and precocial birds overlap, for all values of the slope *b*. Unfortunately the Werner and Griebeler data set [3] does not state which model each of the growth data points uses, so I cannot perform a similar check there.

This speculation is no substitute for a real analysis, however. If it is correct that there is little difference between *M* and *BMatMG*, then what is the value of using *BMatMG* rather than *M*? If incorrect, and the choice does produce large differences in results, that is exactly what a sensitivity analysis would reveal. The published data set lacks the information needed for other researchers to perform such an analysis, but it would be useful to the community if Werner and Griebeler would do so. In the context of the formal comment [1] it is unfortunate that it criticizes Myhrvold [2] for its treatment of the *BMatMG* versus *M* issue without doing the sensitivity analysis that [2] explicitly calls for. That would be a much more scientifically useful way to examine the issue than what is offered in [1].

Although there is no justification given in [3] other than expressed in the quote above, in the formal comment [1] they offer new justifications. The first is that *BMatMG* occurs at the same time in the animal’s growth [14]:

Thus, BMatMG is closer to the maximum growth ability of an individual than its asymptotic mass. Gmax is attained at size BMatMG by the individual, and captures growth conditions at that time. Contrary, the asymptotic size M integrated growth and respective conditions over time (e.g. no growth under food shortage, under starvation animals can even shrink in mass).

The reasoning is problematic for several reasons. The first is that in most situations (including all of the per-taxon data in [3] and [14], estimates of *BMatMG*, *kC*, *M* and *G*_max_ are estimated *together* as part of the model fitting process.

In general, growth curve fits are the preferred method for making such estimates, as Werner and Griebeler recognize [3]:

Today, a proper method for estimating growth rates is to fit non-linear growth functions to growth data. The most commonly used growth models describing individual growth are the Logistic, Gompertz or von Bertalanffy growth functions [12–19].

In analysis based on growth curves a taxon is represented by a set of data points (*t*, *M*(*t*)) where *t* is the age of a specimen, and *M*(*t*) is the body mass of the specimen at age *t*, for one or more individual specimens. In longitudinal data sets there are multiple such points for each individual; cross-sectional data sets have one per individual. These data are used to fit a growth curve which then determines the model dependent growth constant *k*, and the model dependent asymptotic mass *M*. From these one can derive a mass and maximum growth rate data point (*M*, *G*_max_) or (*M*, *kG*) or (*BMatMG*, *G*_max_); these relations are published many places, but appear in [2] as Eqs (1)–(5). Note that some growth models have additional parameters besides *k*, *M*, but the extension to those models is straightforward (see [2,72] and references therein).

Using growth curves allows us to estimate *G*_max_ and/or *kC* (and for that matter *BMatMG*) even if the peak growth occurs in between data points, which is virtually always the case [72]. It also allows us to statistically damp out noise in the individual measurements to see the overall growth pattern and thereby get a better estimate. This is what allows us to cope with the vicissitudes of an animal’s environment during growth including the availability of food or starvation mentioned in the quote.

Because growth models estimate *k*, *M* (and thus *BMatMG*, *G*_max_) simultaneously it is false that *G*_max_ or *BMatMG* “capture growth conditions at the time” whereas *M* is “integrated”. When a growth model is fit to growth data *all* of the parameters are determined simultaneously. The dependence of the parameter estimates on the data point are is global in nature, in the sense that *all* parameters depend in principle on *all* of the data points in the growth series being modeled. This occurs because model fitting minimizes the square of deviation from all data points. Thus all of the data points have an impact on *k*, *M* regardless of whether they are early or late in life, or near the inflection point when maximum growth rate occurs.

The statement quoted above to the contrary shows a conceptual error. The formal comment [1] mistakes the intuitive definition of *BMatMG* and *G*_max_ as different measures (mass and mass rate) that occur together during development, with the mathematical reality of what their study [3] actually did (i.e. determine *M*, *BMatMG* and *G*_max_ simultaneously by a growth model fit).

A second and independent reason to doubt the “at the time” line of reasoning was pointed out by the formal comment [1] (albeit in the context of a different point), which states:

altricial birds and mammals show most of their growth when endothermy is not fully developed [66–69].

This is an important point with regard to preferring “at the time” assessments; if that is the goal then these cases should be classified as ectothermic, which would upend the Werner and Griebeler analysis.

A second argument offered in the formal comment [1] in favor of BMatMG is this:

For fossils, BMatMG is better suited to establish scaling relationships on maximum mass gain than asymptotic size (M). When we have a good mass estimate for the fossil under study, its BMatMG is always documented in its growth record together with Gmax, irrespective of whether a good estimate on its asymptotic mass is available, too (i.e. the animal is fully-grown or a growth model was successfully applied to the specimen in order to estimate M).

Multiple misconceptions are present in this passage. First, there is nothing specific to fossils in this statement or what follows; the mathematics of growth models is the same regardless of the source of the data. Second, if the growth data series are too incomplete or sparse to support a good estimate of *M* they almost certainly do not have a good estimate of *G*_max_ or *BMatMG*.

There is no requirement that every fossil growth series has a data point (*t*, *M*(*t*)) which sits precisely at the inflection point *t*_inf_ so that *M*(*t*_inf_) = *BMatMG*. This is certainly not the case for the dinosaur data sets used in [3] as clearly discussed in [72] which analyzes growth data from the studies that were the source references for [3]. Very few dinosaur growth series include a full range of data point from very young to very old, and most lack data points near the inflection point. All estimates from growth series data that do not sample the full lifespan make all estimates *k*, *M*, *BMatMG*, *G*_max_ uncertain, which was pointed out in Myhrvold [2].

The formal comment [1] poses a hypothetical situation in which a pair of values (*BMatMG*, *G*_max_) is known for a taxon *without* having enough data for a growth curve reconstruction. This is quite unrealistic–in the scenario where there are very few growth data points, how can one be certain that the growth point you have is the true maximum?

This is driven by the geometry of sigmodal growth curves. The point of maximum growth rate (also called the inflection point) occurs in the middle phase of a sigmoidal growth curve. This is called the “linear” stage because growth is approximately linear in time [72], and exactly linear at the inflection point; *G*_max_ is the slope of a line tangent to the growth curve at the inflection point. It is relatively easy to estimate *G*_max_ for growth series that contains two (but ideally three) or more points that are in the linear phase; the slope of a linear regression through those points works well. However the linearity of that phase also makes it very hard to estimate the inflection point, and thus very hard to estimate *BMatMG*. This is particularly true for fossils that have annual growth lines (LAGs–see [72] and references therein). The annual growth increment near the inflection point will be approximately same (due to the linearity of that phase), and will have some measurement error and noise superimposed on the true growth signal. With enough points the location of the maximum can be determined, but with that many points you can also very likely fit the growth curve.

In any event it seems clear that this scenario is both rare and unrealistic. It also *cannot* be the reason *BMatMG* is used in Werner and Griebeler [3] because *none* of the dinosaur growth series in that paper match this hypothetical scenario. Instead this must be a more recent justification in [1].

## 10. Temperature and metabolism

The MTE covers both the scaling of metabolism with body mass which has been discussed in relationship with Werner and Griebeler [3] in a section above. However MTE also covers the scaling of metabolism with body temperature.

$$B_{0}(T)=B_{0}(T_{0})\;exp\left(\frac{E_{i}(T-T_{0})}{k_{B}\;T\;T_{0}}\right),$$ (5)

where *T*_0_ is a normalization temperature, *T* is body temperature, *k*_B_ is Boltzmann’s constant and *E*_*i*_ is activation energy [69,73]. Eq (5) when combined with Eq (4) constitutes the main content of MTE. Eq (5) was termed UTD for “universal temperature dependence” in the original paper [73], but it is also known as the “Boltzmann term” [39,60,74,75], “Arrhenius term” [74,76,77] or “Boltzmann-Arrhenius term” [77] in other works. The formal comment [1] calls it the “Arrhenius term”; here it will be referred to as Eq (5). The reason for these names is that Eq (5) is superficially similar to the Arrhenius rate reaction equation from chemistry, and the Boltzmann distribution from thermodynamics. Note that (5) does *not* automatically follow from these earlier relations [48,78], and that adherence to (5) across organisms is a statistical correlation at best [78].

This relationship is the background to a set of criticisms raised in the formal comment [1] which are more relevant to Grady *et al*. [14] than to Myhrvold [2]. Grady *et al*. [14] includes maximum growth rate and body mass data for many species and taxa (i.e. (*M*, *G*_max_) for each taxon). They also employ basal or resting metabolic rate data for some of the taxa.

Myhrvold [2] used a variable named *BMR* to describe this, and described it as a basal metabolic rate. As Griebeler has noted, basal is a term defined for endotherms, while resting metabolic rate is a more appropriate term for the same concept for ectotherms. The looser sense of “basal metabolic rate” for both endotherms and ectotherms does however appear in textbooks [69], and to be consistent with Myhrvold [2], I continue that usage here.

Ectotherm metabolic rates are sensitive to body temperature, which in turn is highly dependent on ambient environmental temperature. In their supplemental online information, Grady et al. say this about how they handled differing ambient temperatures [14]:

We have standardized ectotherms temperatures to an ambient temperature of 27°C to facilitate comparison with dinosaurs, but our results are not qualitatively affected by variation in standardized temperature between 25–30°C (S2 Fig).

The formal comment [1] criticizes this as “flawed”. And it may be, but whether it was flawed or not was unimportant for Myhrvold [2], which used the *BMR* data as presented in Grady *et al*. [14], for three reasons.

First, the second author of Grady *et al*. [14], Brian Enquist, is one of the authors on the original paper introducing MTE [68] and has been deeply involved in its development and application since then [79–81]. Second, the sensitivity analysis of S2 Fig of Grady *et al*. [14] (in its supplementary information) seems convincing that across a wide range of temperatures the results were likely not to be effected too much by their choice of standard temperature. Although both of these points could be incorrect, the benefit of the doubt seemed warranted.

The third and overwhelmingly most important reason to use the Grady *et al*. *BMR* data without modification was that Myhrvold [2] criticized those authors for internal contradictions in their arguments, by showing that their own data did not match their conclusions. Such criticisms are less persuasive, and more likely to be in error, if they are based on readjusted data.

The GW comment criticizes results in Myhrvold [2] derived from the Grady *et al*. *BMR* data. One such result concerns Fig 7 of [2] which shows that taken together *M*, *BMR*, *kC* are a specific example of the intransitivity of statistical correlation. The figure shows that *M*, *BMR* are correlated, and *M*, *kC* are correlated, but despite this *BMR*, *kC* have essentially no correlation. This is what one would expect from mathematics (see [2] and references therein) but is quite counterintuitive. It also demonstrates that hypothesis H2 is not correct.

The formal comment [1] argues that this does not show H2 is incorrect, because the Grady *et al*. *BMR* data, or its temperature adjustment, are flawed. Yet hypothesis H2 was formulated specifically to address the results of Grady *et al*. linking metabolic rate to maximum growth rate (specifically their Eqs 1–3 and surrounding text). It is these results which Grady *et al*. had supported with their *BMR* data (for example, their Fig 3 and others in their supplemental information). In the context discussed above, Myhrvold [2] demonstrated that the Grady *et al*. *BMR* data did not actually support the results claimed.

The formal comment [1] argues that hypothesis H2 and by extension Grady *et al*. Eqs 1–3 need a temperature correction term, which appears in their Eq (2). It is only if temperature effects on maximum growth rate and metabolism can be ignored that H2 holds (their Eq (3)). Grady et al. [14] approached this by making an overall temperature correction for ectotherms, thus eliminating temperature from their statement of what I later termed H2 in [2].

In effect, the formal comment [1] argues that Grady *et al*. should have proposed a *different* link between maximum growth rate and metabolism (i.e. their Eqs (2) and (3))—a version of H2 which includes a temperature correction. Moreover, the formal comment [1] argues that I should have adjusted Grady *et al*. *BMR* data by per-species temperature data (rather than data standardized to a single temperature) to disprove that temperature-enhanced H2.

They are entitled to their opinion, but the fact is that Grady *et al*. made the proposal that they did, *not* the one that the formal comment [1] suggests they should have made. And I argued against it using *their data set as they used it*. If Griebeler and Werner wish to propose their version of a temperature corrected H2, then they can publish a paper to that effect. In Myhrvold [2] I was not critiquing that hypothetical work, I was focused on [3] and[14].

Alternative, Griebeler and Werner have come up with an independent argument as to why the Grade et al version of H2 is incorrect. Either way it seems misplaced to direct their criticism at [2] which simply disproves Grady et al with their own data.

The other use of the Grady *et al*. *BMR* data in [2] is Fig 6 of [2]. This figure plots the residuals after fixed slope 0.75 regression—i.e. what Werner and Griebeler [3] term residual variation (i.e. Eq (3) above.) Fig 6 of [2] plots this for *kC*, and for the Grady *et al*. *BMR* data. These plots are quite different. The residual variation plots for *kC* show considerable overlap between endotherms and ectotherms (just as Figs 2 and 3 above do), while the residual variation for *BMR* shows a separation between endotherms and ectotherms. If instead of *kC* I had plotted *G*_max_, the result would be qualitatively similar with considerable overlap between endotherms and ectotherms.

The formal comment [1] has this criticism:

The clear separation in the scaling regressions on MR observed between endothermic and ectothermic species by Myhrvold [10] is a shear temperature effect in the raw data of Grady et al. [7] that he adopted for his analysis. Body temperatures of mammals and birds (e.g. range in mammals: 30.4–40.1°C, range in birds: 37.5–44.6°C; from Griebeler [54]) are considerably higher than the 27°C to which these authors adjusted resting metabolic rates of ectothermic species. We thus expect the separation of regression lines on MR against M (sixth figure in Myhrvold [10]) simply from the temperature dependence of any chemical reaction.

In effect, they argue that the *BMR* is simply a reflection of body temperature, so the clear separation in Fig 6 of [2] is simply due to body temperature. There are two independent questions this raises. The first being: how are they sure? Unless they plot the Grady et al. data with the methods that they argue are correct and show that it is all due to body temperature there is no actual proof just a claim. Indeed in the formal comment [1] they say this about Eq (5):

All these observations question the MTE as they indicate that differences in body temperature are not sufficient to fully explain differences in Gmax and MRs of similar-sized growing ectotherms and endotherms [54].

If body temperature differences are not sufficient via MTE (i.e. Eq (5)) to explain the differences in metabolic rate, then how can they be sure that the “shear temperature effect” completely explains Fig 6 of [2]?

The second and primary question is: why would this matter? The formal comment [1] claim, *even if true*, is quite irrelevant to the point that Fig 6 of [2] is making: which is that *BMR* acts very differently than maximum growth rate when adjusted for mass with slope *b* = 0.75 (essentially the same procedure as Eq (3) here.) It is entirely valid to make that point using the Grady et al. data set. The fact that body temperature may explain some, or all, or none, of the difference in metabolism is simply not relevant to Fig 6 of [2], or what it is trying to show. This is directly relevant to the Grady et al. hypothesis about *BMR* and *G*_max_ (their Eqs (1)–(3)), and so was appropriate for [2]. Note in Fig 6 of [2] the maximum growth rate plotted is *kC*, but *G*_max_ would act similarly.

At best the formal comment [1] is making an argument to explain the origin of the difference–i.e. attempting to explain why *BMR* varies between endotherms and ectotherms, and why I find a clear separation. Explaining that body temperature is underlying factor in metabolic rate does not in any way invalidate the point that Fig 6 of [2] is making.

There is a longstanding debate in biology as to whether body temperature drives metabolism, or the other way around, or something in between [37,61,69,82–85]. Even if one accepts Eq (5) above, it can be inverted to yield temperature as a function of metabolic rate. Indeed the thermoregulation strategies of endotherms could be viewed as a means to force the causality in the reverse direction, using physiological means to generate and support body temperature [24,35,47].

Finally it is worth noting that Eq (5) is an integral part of MTE. The formal comment [1] quoted above argues that they “question the MTE”, and go on to specifically question the temperature dependence (see the quoted passage in section 7 above)–i.e. they appear to have a problem with Eq (5). Yet in arguing that Myhrvold [2] and Grady et al. [14] do not do temperature correctly properly, they seem to fully support Eq 5 –to the degree that they believe one of the originators of MTE is applying it incorrectly (i.e. in Grady et al. [14]). This appears to be remarkably inconsistent.

The formal comment [1] states that its authors “wonder” why I mention Werner and Griebeler [3] in connection with hypothesis H2 (see section above). Yet they also defend it argue that the results refuting H2 in [2] were not correct, so that I haven’t actually disproven it (or disproven the temperature effect version of H2 they argue it should be). This is another seeming inconsistency in their position.

## 11. Group composition

The final issue to discuss is the composition of groups used by Case [4,5], the dinosaur growth papers that cite Case [6–13], Werner and Griebeler [3] and Grady et al. [14]. Although there are some differences in the groups across these papers, they are broadly similar and appear to be either directly based on Case, or small extensions of the Case groups. Case followed the custom of the biological literature of his time in choosing groups which reflect common usage division of vertebrates (i.e. “fish”, “reptiles”) rather than phylogenetically precise groups which map directly to clades. This was sufficient for the purposes that Case had.

The papers which promote or use H1 [6–13] explicitly assume that the regression parameters *a*, *b* for a group are a reason to classify the metabolism of all members of the group. If the group involved is a clade–i.e. it is monophyletic–then regression across the clade produces the average behavior of the clade. Using a phylogenetically informed regression like PGLS, or PIC (used in Grady et al. [14]) we would expect this average to approximate the shared phylogenetic heritage of the group (as embodied as its most recent common ancestor) [78,86–89]. If the group is paraphyletic then it would be some approximation of group-wide behavior and common ancestry. In either case the regression parameters are tied to inherited features–i.e. the purported link between metabolism and maximum growth rate.

However some of the groups are polyphyletic. Regression across a polyphyletic group is explicitly not about lineage, and instead is about convergent evolution across whatever shared features define the group. As an example, precocial birds are a polyphyletic group [49].

As discussed in Myhrvold [2], it is unusual to combine lineage based and convergent based groups. The lineage oriented groups are treating the growth rate to metabolism link as an inherited trait, while the other groups treat the link as a labile trait. It is not impossible for it to be both but it an unusual combination, made all the more so by the fact that the convergent based groups are idiosyncratic–birds are divided into altricial and precocial, while eutherian mammals which also have altricial and precocial members are not.

This is an inherent aspect of any regression–the assumption in a regression analysis is that there is a correlation between the independent and dependent variables which is obscured by some amount of uncorrelated random noise. The regression yields an estimate of the average behavior of the variables across the group that it covers; this average behavior is dependent not only on the variables, but also the choice of the group (data set) covered by the regression. In a lineage based group there is an implicit assumption that this average behavior is a property of the lineage (although it may not be). In a polyphyletic based group the assumption is that lineage does not matter (because there is no common shared lineage); indeed lineage can be part of the “noise” in such a case.

Each is also problematic for the purposes that H1 puts the groups to. In a paraphyletic group like the dinosaur group in [6–13], which spans a huge tree and more than a hundred million years why must we believe that all members have the same metabolism [2]? If the dinosaur group were made monophyletic (i.e. birds are added) then the results change (there would be overlap between that group and its closest outgroups–reptiles and mammals). More generally in monophyletic groups (eutherian mammals, marsupials), the average behavior from regression is, at best, telling us something about traits shared across the lineage–i.e. those of the most recent common ancestor, *not* the diversity that has evolved since then. Also, any method of classifying all members of a group by the group-wide average falls under the ecological fallacy. These points were made in Myhrvold [2].

This criticism applies to the analysis in Werner and Griebeler [3] to the extent that is interpreted like H1 (see above). It also applies in a much weaker form to the residual analysis as it is portrayed in [3] as being dependent on the regression parameter *a*. The issues with *a* are essentially the same as those discussed above for *a*, *b*. However that is how the analysis is *described* in [3], as shown above in Eq (3) the actual analysis is independent of regression parameter as shown above, so it avoids the ecological fallacy. As a result, group composition does not matter much to how [3] actually performs its analysis. In an operational sense the groups that matter to the residual variation analysis are endotherm versus ectotherm. It is portrayed as also depending on synapsid versus not because of the restriction to synapsids discussed above, but since synapsid endotherm and ectotherm growth rates overlap there is no distinction in practice.

## 12. Conclusions

Werner and Griebeler made an important contribution to the field by publishing the first serious analysis of the question of whether dinosaur metabolism can be determined from growth-rate allometry [3]. My subsequent analysis [2] confirmed one of their conclusions: that one cannot classify the metabolism by comparing growth-rate allometry to extant groups. I commended them in [2] for this, and I do so again here.

Unfortunately their paper was also marred by an inconsistent and incorrect insistence that dinosaur metabolism can nonetheless be determined from growth-rate allometry—because, they argue, one must ignore the general vertebrate case and restrict the analysis to sauropsids. This is problematic at multiple levels.

First, their original paper [3] never offered an explanation for why the restriction to sauropsids is valid. Their formal comment does suggest new arguments. Unfortunately, each is flawed, for reasons explained in Sections 1 and 2.

A second issue with their conclusion about dinosaur metabolism is its reliance on an incorrect empirical result, namely that growth rates for endothermic and ectothermic sauropsids in their data set do not overlap. Their own data and a figure in their paper [3] clearly shows that they do in fact overlap (Fig 3 and Table 1). Additional data from Grady et al. [14] corroborates this (Table 1). The formal comment [1] is clear that any overlap “would reject our conclusion” that dinosaurs are ectotherm. I concur with that assessment.

Much of the formal comment [1] delves into tangential questions of methodology that have little to no relevance to the main results of [3] or their treatment in [2]. Examination of these criticisms finds that most are not warranted. A complaint that Myhrvold [2] used OLS rather than PGLS regression fails to recognize that the analysis in [3] effectively canceled out the effect of the regression (see Eq (3)), making the choice of regression method irrelevant, at least for the residual variation overlap analysis that drives their main conclusion. The equivalence of OLS and PGLS is apparent in the residual variation plots they presented, which were essentially identical (Fig 2). Similarly, a discussion contrasting the *kC* approach versus the *G*_max_ approach is undermined by the fact that the two approaches are mathematically identical. The counterexample proposed in the comment is shown to be trivially irrelevant.

Absent a sensitivity analysis or a fuller explanation by Werner and Griebeler, it is unfortunately not possible to resolve the question of whether the choice of *BMatMG* vs. *M* as independent variable affects the final results of [3] in any qualitative way. The comment offers new arguments, but these rely on misconceptions about growth-model fitting. The critique of temperature adjustment for ectotherms in Grady et al., and the role of temperature dependence in the MTE are also found to be flawed, as well as inconsistent with the authors claim that they are skeptical of the MTE. The final issue, regarding group composition critiqued in [2], largely does not apply to [3] because the results of that study do not depend on regression.
